# Morphology of the medial collateral ligament of the knee

**DOI:** 10.1186/1749-799X-5-69

**Published:** 2010-09-16

**Authors:** Fang Liu, Bing Yue, Hemanth R Gadikota, Michal Kozanek, Wanjun Liu, Thomas J Gill, Harry E Rubash, Guoan Li

**Affiliations:** 1 Bioengineering Laboratory, Orthopaedic Department, Massachusetts General Hospital, Harvard Medical School, 02114 Boston, USA; 2 Department of Orthopaedic Surgery, Shanghai Ninth People's Hospital, Shanghai Jiaotong University School of Medicine, Shanghai, China; 3 Department of Orthopaedic Surgery, Yantaishan Hospital, Yantai, China

## Abstract

**Background:**

Quantitative knowledge on the anatomy of the medial collateral ligament (MCL) is important for treatment of MCL injury and for MCL release during total knee arthroplasty (TKA). The objective of this study was to quantitatively determine the morphology of the MCL of human knees.

**Methods:**

10 cadaveric human knees were dissected to investigate the MCL anatomy. The specimens were fixed in full extension and this position was maintained during the dissection and morphometric measurements. The outlines of the insertion sites of the superficial MCL (sMCL) and deep MCL (dMCL) were digitized using a 3D digitizing system.

**Results:**

The insertion areas of the superficial MCL (sMCL) were 348.6 ± 42.8 mm^2 ^and 79.7 ± 17.6 mm^2 ^on the tibia and femur, respectively. The insertion areas of the deep MCL (dMCL) were 63.6 ± 13.4 mm^2 ^and 71.9 ± 14.8 mm^2 ^on the tibia and femur, respectively. The distances from the centroids of the tibial and femoral insertions of the sMCL to the tibial and femoral joint line were 62.4 ± 5.5 mm and 31.1 ± 4.6 mm, respectively. The distances from the centroids of dMCL in the tibial insertion and the femoral insertion to the tibial and femoral joint line were 6.5 ± 1.3 mm and 20.5 ± 4.2 mm, respectively. The distal portion of the dMCL (meniscotibial ligament - MTL) was approximately 1.7 times wider than the proximal portion of the dMCL (meniscofemoral ligament - MFL), whereas the MFL was approximately 3 times longer than the MTL.

**Conclusions:**

The morphologic data on the MCL may provide useful information for improving treatments of MCL-related pathology and performing MCL release during TKA.

## Introduction

Medial collateral ligament (MCL) consists of two components, the superficial MCL (sMCL) and deep MCL (dMCL). The MCL has been described as the primary static stabilizer against valgus rotation of the knee joint [1,2]. In total knee arthroplasty, soft tissue balance of the varus knee always requires partially releasing the MCL for achieving proper knee alignment [3-6]. Quantitative knowledge of the MCL anatomy is therefore critical for improvement of surgical procedures that involve the MCL complex.

Most studies on MCL anatomy have been conducted by dissection of cadaveric human knees [7-11]. Ligament lengths and insertion areas have been generally reported [11]. The sMCL has usually been described as a ligament connecting the medial tibia and femur and has been investigated in greater detail than the dMCL. However, there is discrepancy in the literature on the femoral attachment of the sMCL. The dMCL has been divided into two portions, the proximal half (meniscofemoral ligament - MFL) and the distal half (meniscotibial ligament - MTL) [11,12]. Little data has been reported on the anatomic features of the dMCL. A thorough understanding of the sMCL and dMCL anatomy may provide baseline knowledge for surgical management and further research.

The objective of this study was to quantitatively determine the morphology of the MCL of human knees through an anatomic dissection of human cadaveric knees. Specifically, we determined the ligament lengths, insertion areas, and distance with respect to the meniscus as well as the tibiofemoral joint line with the knee installed in a full extension position.

## Materials and methods

### Cadaveric Knees

Ten fresh frozen, unpaired cadaveric knees having no evidence of pathology or damage were utilized for this study. The mean age of the donors was 61.2 years (57 to 64). Each cadaveric knee was fresh frozen at -20°C and thawed overnight prior to dissection. Each knee was transected ~30 cm above and below the joint line. The shafts of the tibia and femur of each specimen were potted in thick walled aluminum cylinders using polymethylmethacrylate cement. The specimen was then aligned in full extension that was maintained during the dissection and morphometric measurements.

### Anatomic Measurements

**Figure 1:**
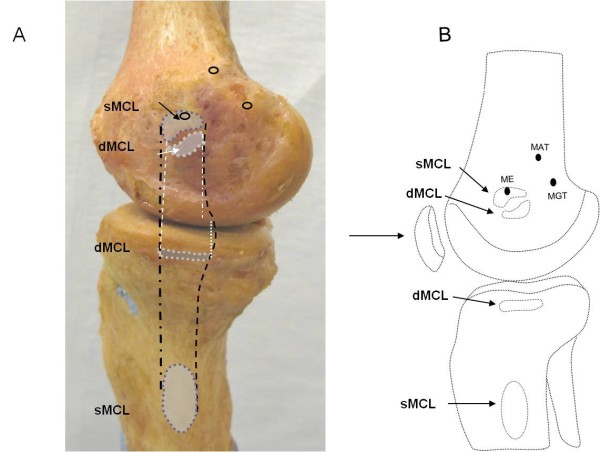
**Medial aspect of the knee**. (A). Photograph showing the medial side of the right cadaveric knee joint. (B). the distances from the centroid of femoral insertion and tibial insertion to the joint line. sMCL = superficial medial collateral ligament, dMCL = deep medial collateral ligament, POL = posterior oblique ligament. ME = medial epicondyle, MGT = medial gastrocnemius tubercle, MAT = medial adduct tubercle.

**Figure 2:**
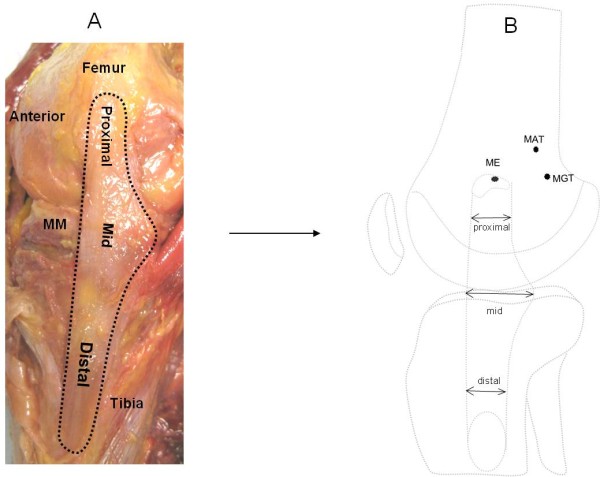
**Dissected insertions of the MCL**. (A). Photograph showing the three parts of the sMCL (right cadaveric knee). (B). Schematic diagram illustrating the width of sMCL at the proximal, middle and distal parts.

**Figure 3:**
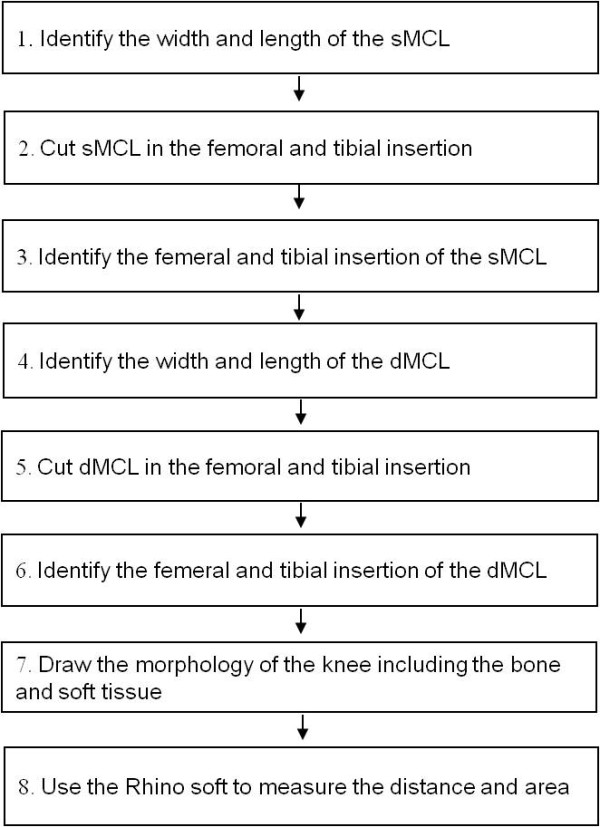
The experimental sequence.

**Figure 4:**
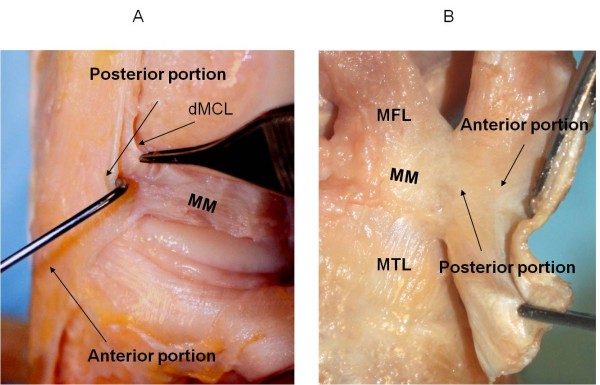
**Posteromedial corner of the knee**. (A). Photograph showing the posterior portion of the sMCL was firmly attached to the medial meniscus of the knee (right knee); (B). the anterior portion of the sMCL was cut and everted, the posterior portion of the sMCL was connect to the meniscus.

The insertion sites of the sMCL and dMCL were identified from their bony insertions of the femur and tibia (Fig. 1A). Three anatomic landmarks on the femur (ME = medial epicondyle, MGT = medial gastronomies tubercle, MAT = medial adductor tubercle) were specified as references for later measurements (Fig. 1B)[11]. The sMCL and dMCL were separated by a bursa in all cadaveric knees. The sMCL was carefully separated from the dMCL without damaging the dMCL during the dissection. The anterior part of the sMCL was vertically aligned but the posterior part was oblique (Fig. 2A). Unlike the anterior part, the posterior part of the sMCL was found firmly attached to the medial meniscus (Fig. 3)[13]. The dMCL, which consisted of a proximal portion (meniscofemoral ligament - MFL) and distal portion (meniscotibial ligament - MTL), was relative thinner compared to the sMCL (Fig. 4A). The *pes anserinus *tendons (sartorius, gracilis, and semitendinosus tendon) were detached from their tibial attachments during dissection. A fine-point marker was used to outline the location of the medial structures of the knee.

The outlines of the insertion sites of each ligament were then digitized using a 3D digitizing system which has a reported accuracy of 0.3 mm (MicroScribe G2LX; Immersion Technologies, San Jose, CA, USA). The digitized points were imported into solid modelling software (Rhinoceros; Robert McNeel and Associates, Seattle, WA, USA) to calculate the areas of the insertion sites and the centroids of the insertion areas. These values were calculated by using the inbuilt functions ("Area" and "AreaCentroid") of the Rhinoceros software.

**Figure 5:**
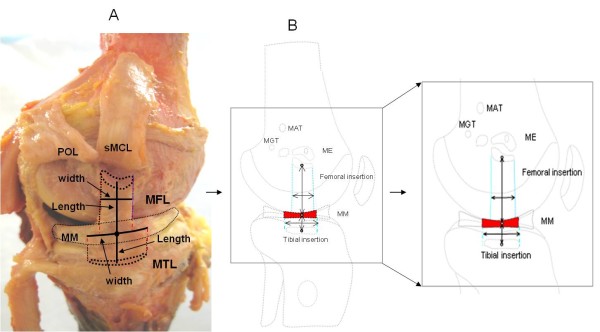
**Dissected deep medial structures of the knee.** (A). Cadaveric view of the femoral and tibia attachment of medial structures of the knee (left knee); (B). Schematic diagram illustrating the length and width of the meniscofemoral ligament and meniscotibial ligament. MM = medial meniscus, MFL = meniscofemoral ligament, MTL = meniscotibial ligament.

In this study, we first determined the insertion areas of the sMCL and dMCL on the femur and tibia (Fig. 1A). We then measured the distances between the centroids of the insertion areas to determine the length of the ligament [11]. Joint line was determined according to the previous definitions of Laprade et al. [11], where the edge of the articular cartilage surface of the medial femoral condyle was defined as the femoral joint line and the medial tibial plateau as the tibial joint line. All measurements were performed according to a sequence of eight steps (Fig. 5).

### Statistical analysis

A one-way repeated-measure ANOVA was used to compare the width of the sMCL among the proximal, mid and distal parts. A paired Student t-test was used to compare the results for lengths and widths of the dMCL, and the insertion areas of sMCL and dMCL. Differences were considered statistically significant when P < 0.05.

## Results

### Superficial Medial Collateral Ligament

**Figure 6:**
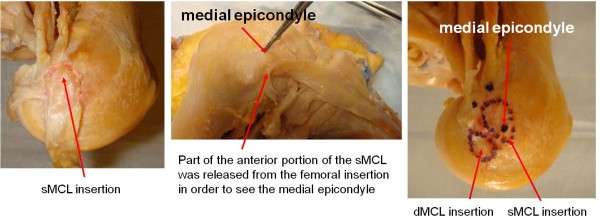
**Femoral insertion and medial epicondyle of the sMCL**. (A). sMCL attachment on the femoral condyle; (B). the medial epicondyle under the anterior poriotn of the sMCL insertion; and (C). bony locations of the sMCL and dMCL insertions and medial epicondyle on the femoral condyle.

The insertion areas of the superficial MCL are listed in Table 1 and Table 2. The femoral insertion of the anterior postion of sMCL covers the medial femoral epicondyle (Fig. 6). The tibial insertion was 62.4 ± 5.5 mm distal to the level of the tibial joint line (Fig. 1B). The insertion areas of the sMCL on the femur and tibia were 79.7 ± 17.6 mm^2 ^(range, 58.8 to 116.1) and 348.6 ± 42.8 mm^2 ^(range, 285.3 to 427.3), respectively. The distance between the centroid of sMCL on its femoral insertion to the joint line was 31.1 ± 4.6 mm (range, 24.4 to 39.8). The average distance between the centroid of the sMCL on its tibial insertion to the tibial joint line was 62.4 ± 5.5 mm (range, 54.7 to 71.5).

**Table 1:** Quantitative measurements of sMCL and dMCL on their femoral insertion and their relationship to the joint line, medial epicondyle, medial gastrocnemius tubercle and medial adduct tubercle

	Femoral insertion			Distance from the centroid of femoral insertion to			
	**Area (mm**^**2**^)	**Width (mm)**	**Length (mm)**	**Femoral Joint line (mm)**	**ME**	**MGT**	**MAT**
	
sMCL	79.7 ± 17.6	11.8 ± 3.4	9.0 ± 1.9	31.1 ± 4.6	2.9 ± 0.8	14.7 ± 4.5	16.5 ± 1.6
dMCL	71.9 ± 14.8	9.9 ± 3.2	9.4 ± 3.3	20.5 ± 4.2	13.0 ± 2.7	22.1 ± 4.6	27.4 ± 5.1

ME = medial epicondyle, MGT = medial gastrocnemius tubercle, MAT = medial adductor tubercle

**Table 2:** Quantitative measurements of sMCL and dMCL on the tibial insertion and its relationship to tibial joint line.

	Tibia insertion			Distance from the centroid of the tibial insertion to
	**Area (mm**^**2**^)	**Width (mm)**	**Length (mm)**	**Tibial Joint line (mm)**
	
**sMCL**	348.6 ± 42.8	14.9 ± 5.7	31.1 ± 8.1	62.4 ± 5.5
**dMCL**	63.6 ± 13.4	18.0 ± 4.0	5.1 ± 1.8	6.5 ± 1.3

The mean overall length of the sMCL measured from the centroid of the femoral insertion to the centroid of its distal tibial insertion was 100.7 ± 9.5 mm (range, 90.0 to 117.1). The sMCL was broad, flat and triangular in shape (Fig. 2A). Therefore, in order to identify the actual morphology of the sMCL, it was theoretically divided into three parts, the proximal, mid and distal sMCL. Those were measured as 10.9 ± 1.2 mm, 17.7 ± 2.1 mm and 10.7 ± 1.8 mm, respectively, in the anterior-posterior direction (Fig. 2B) Table 3. The average width of the sMCL in the proximal and distal part was similar (P = 0.99). The average mid width, which is the widest and firmly attached to the medial meniscus in its posterior portion (Fig. 3), was 1.6 times wider than the proximal or the distal part (P < 0.05). The distances from the centroid of the femoral insertion of the sMCL to the medial epicondyle, medial gastrocnemius tubercle and adductor tubercle were 2.9 ± 0.8, 14.7 ± 4.5 and 16.5 ± 1.6 mm, respectively (Table 1).

**Table 3:** Quantitative measurements of the sMCL and dMCL (MFL/MTL) in width and length (mm)

	sMCL			dMCL	
	**Proximal (mm)**	**Mid (mm)**	**Distal (mm)**	**MFL (mm)**	**MTL (mm)**
	
**Width**	10.9 ± 1.2 (8.5 to 12.3)	17.7 ± 2.1 (14.6 to 20.1)	10.7 ± 1.8 (8.1 to 14.4)	10.6 ± 4.5 (5.2 to 19.5)	17.9 ± 2.7 (14.8 to 22.8)
**Length**	100.7 ± 9.5 (90.0 ± 117.1)			26.2 ± 5.6 (16.5 to 34.0)	9.2 ± 1.8 (7.2 to 11.8)

### Deep Medial Collateral Ligament

The femoral insertion of the dMCL was located below the insertion of the sMCL (Fig. 1). The quantitative measurements of insertions of the dMCL on the femur and tibia are listed in Table 1 and Table 2. The length of the MFL was measured from the centroid of its femoral insertion to the medial centroid of the medial meniscus that was attached to the dMCL (Fig. 4). The length of the MTL was defined from the centroid of its tibial insertion to the center of its insertion on the medial meniscus. The insertion areas of the dMCL on the femur and tibia were 71.9 ± 14.8 (range, 45.9 to 96.7) and 63.6 ± 13.4 mm^2 ^(range, 39.6 to 88.5), respectively. The average distance between the centroid of its femur insertion of the dMCL to the femoral joint line was 20.5 ± 4.2 mm (range, 15.0 to 29.4) (Table 1). The average distance between the centroid of dMCL on its tibial insertion to the tibial joint line was 6.5 ± 1.3 mm (range, 4.2 to 9.5) (Table 2). The distances from the centroid of its femoral insertion to the medial epicondyle, gastrocnemius and adductor tubercle were 13.0 ± 2.7, 22.1 ± 4.6 and 27.4 ± 5.1 mm, respectively (Table 1) (Fig. 4B).

## Discussion

The study quantitatively determined the morphology of the MCL (sMCL, dMCL). The ligament length and its insertion areas on the femur and tibia were measured upon dissection of cadaveric human knees in full extension. We found that the sMCL was triangular in shape and the proximal and distal parts were composed of parallel fibers, whereas the middle part of the sMCL was composed of parallel and oblique fibers. We found that the widths of proximal and distal parts were similar in the anteroposterior direction; the width in the middle part was 1.7 times wider than the proximal/distal parts (P < 0.05). The anterior portion was not attached to the medial meniscus and could be distinguished from the capsule of the knee joint. However, our dissection found that the posterior portion was firmly attached to the medial meniscus (Fig. 3A, B).

To our knowledge, there are currently no reports quantifying and statistically comparing the two individual components of the MCL. The overall length of the sMCL measured in this study was similar to those of previous reports [7,8,11]. LaPrade et al.[11] showed that the distal tibial insertion area is larger than the femoral insertion area. Further, our measurements of the distance from the femoral and tibial insertions of the sMCL to the joint lines, in general, are consistent with the data reported previously by others[11,14]. However, there are certain variations in description of the location of the femoral insertion of the sMCL in literature [8,11,13,15-19]. Some reports state that the femoral insertion of the sMCL was located on the medial epicondyle of the femur [8,13,15,17-19] while others report that the femoral insertion site of the sMCL was located slightly proximal to the medial epicondyle [11,16]. We found that the anterior portion of the sMCL femoral insertion covered the medial epicondyle as shown in Fig. 6. Our results also confirmed the previous assumption by Brantigan et al., who stated that the oblique portion of the sMCL was indistinguishable from the true capsule, and might be considered attached to the medial meniscus [16]. Last el al. also observed that the posterior part of the superficial medial ligament is attached to the medial meniscus [13]. It is important to recognize that the posterior portion of sMCL is attached to the medial meniscus. Since the meniscus is removed during TKA the function of the sMCL may be affected even without further soft tissue releasing.

The sMCL plays a critical role in the success of total knee arthroplasty (TKA). Appropriate soft tissue balancing has a direct effect on the knee joint function after TKA [3,5,6,20]. Partial releasing of the sMCL has often been performed for joint alignment. Releasing the sMCL alters the functional capacity of the ligament. More, the dMCL which attachment is on average about 6 mm distal to the tibial plateau is often released during TKA further weakening the medial side of the knee. However, the clinical importance of this remains undetermined. Future research needs to quantitatively determine how the soft tissue releasing, although beneficial for joint alignment, affects the knee joint function after TKA.

Fewer quantitative data has been reported on the morphology of the dMCL as compared to the sMCL. The dMCL anatomy has been analyzed into two parts, i.e. MTL and MFL[9,19,21]. LaPrade et al. described that the MFL was longer than the MTL. Similar to the findings of this study, the MFL was found to be approximately three times longer than the MTL (P < 0.05). They also reported the distance from the tibiofemoral joint line to the MTL tibial attachment was on average 3.2 mm (1.8 to 5.9), which was lower than the distance measured in this study (6.5 mm). The MTL was approximately 1.7 times wider than the MFL (P < 0.05), whereas MFL was approximately 3 times longer than the MTL (P < 0.05). At the femoral insertion, a bursa was found between the sMCL and dMCL, similar to the report by Sims et al. [19].

The dMCL was firmly attached to the medial meniscus at the joint line. The dMCL might play an important role to anchor the peripheral parts of the medial meniscus in the medial side of the knee (Fig. 3). In a clinical report, the MTL injury was found to be more common than that of the MFL. The MTL insufficiency may contribute to increased stress on other structures, including the meniscus, that resist anterior and anteromedial displacement and can lead to further injury [19]. However, since the dMCL lies deep to the sMCL, diagnosis and surgical repair of a dMCL injury is still a challenge.

This study had several limitations. Laprade et al.[11] reported that the sMCL had two distinct tibial attachments and the proximal tibial attachment was primarily to soft tissue. We observed that the proximal tibial insertion of the sMCL was the soft tissue connection not the bony insertion. In our specimens, the proximal tibial insertion was relatively easy to separate from the attachment site but it was difficult to quantitatively define the outline of the tibial proximal insertion using the dissection method. Therefore, it was difficult to consistently and accurately measure the proximal insertion of the sMCL. In addition, the measured length of sMCL might be less than the actual sMCL due to the straight lines that were used to approximate its length. Finally, all the measurements were made only at knee full extension. Measuring in full extension was chosen because the clinical examination also realies on testing varus-valgus stability in near-extension.

In summary, this study measured the anatomy of the MCL complex by dissection of cadaveric human knee specimens. The lengths, insertion locations and insertion areas as well as relations to medial meniscus were quantitatively measured for sMCL and dMCL. The present data on MCL complex anatomy can provide useful information in performing intraoperative assessment of MCL injury and ligament replacement for the surgical repair or reconstruction of the MCL. Furthermore, the quantitative data regarding the medial structure can have clinical implication during surgical release of the MCL in TKA.

## Completing interests

The authors declare that they have no competing interests.

## Authors' contributions

All authors read and approved the final manuscript.
